# Non-compliance of hand hygiene using covert and overt methodology among healthcare workers at a tertiary care hospital in Saudi Arabia

**DOI:** 10.1186/2047-2994-4-S1-P301

**Published:** 2015-06-16

**Authors:** A El-Saed, S Noushad, HH Balkhy

**Affiliations:** 1 Infection control, King Abdulaziz Medical City, Riyadh, Saudi Arabia

## Introduction

Hand hygiene (HH) is a core element in preventing healthcare-associated infections and the spread of antimicrobial resistance. Although the benefits of HH are non-controversial, the HH compliance is still suboptimal. The degree of HH compliance is thought to be affected by the awareness of healthcare workers (HCWs) of being monitored.

## Objectives

To estimate the frequency of HH non-compliance using covert methodology and to compare such frequencies to corresponding traditional HH surveillance from the same locations and time periods.

## Methods

A cross-sectional study design was conducted at King Abdulaziz Medical City, Riyadh, Saudi Arabia, between October 2012 and July 2013. Non-compliance was defined as missing both hand rubbing (with alcohol-based formulation) and hand washing (with soap and water) during one of the WHO five-moment HH indications (opportunities). Observations were conducted quietly without attempts to promote HH compliance or provide performance feedback to HCWs.

## Results

A total of 6580 opportunities were observed among different professional categories in 28 hospital locations. The overall non-compliance from the current study was 54.1% compared with 12.9% in traditional HH surveillance (p<0.001). The same significant trend was replicated by professional category (53.1% vs. 10.2% in nurses, 53.8% vs. 19.6% in physicians, and 57.1% vs. 13.2% in other HCWs), service locations (54.3% vs. 8.6% in intensive care and step-down units, 51.8% vs. 14.1% in inpatient wards, and 58.2% vs. 16.0% in emergency locations), and HH indications (47.5% vs.10.5% in pre-contact with patients, 60.1% vs.10.5% in post-contact with patients, 57.3% vs.2.7% in post-exposure to body fluids, 68.5% vs.5.6% in before aseptic tasks, and 49.8% vs.17.5% in post-contact with patient surroundings).

## Conclusion

Major differences in non-compliance were detected during covert and overt HH monitoring irrespective of hospital location, professional category, and indications. Overlapping methods of auditing and frequent change of data collectors may reduce the underestimation of non-compliance.

## Disclosure of interest

None declared.

